# Editorial: Decision neuroscience of attention

**DOI:** 10.3389/fnins.2023.1223299

**Published:** 2023-09-06

**Authors:** Soo Hong Chew, Richard P. Ebstein, Serenella Tolomeo, Yuan Zhou

**Affiliations:** ^1^CCBEF (China Center for Behavior Economics and Finance) & SOE (School of Economics), Southwestern University of Finance and Economics, Chengdu, Sichuan, China; ^2^Department of Economics, National University of Singapore, Singapore, Singapore; ^3^Institute of High Performance Computing (IHPC), Agency for Science, Technology and Research (A^*^STAR), Singapore, Singapore; ^4^Institute of Psychology, Chinese Academy of Sciences (CAS), Beijing, China

**Keywords:** attention mechanisms, behavioral neuroscience, bottom-up processes, goal-directed decision making, neuromodulators, salience network, stimulus driven, top-down processes

## Attention is in the air

This Frontiers volume on Decision Neuroscience presents nine articles highlighting the importance of attention as a fundamental mechanism underpinning decision making. A crucial distinction is between goal-directed top-down (TD) and stimulus-driven bottom-up (BU) processes (Connor et al., 2004). This distinction seems to inform Posner and Petersen's (1990) division of attentional processes into alerting, orienting, and executive functions. While the orienting network involves both TD and BU processes, the alerting and executive networks are respectively of a BU and TD nature.

The article by Posner et al. details the role of attention in animal and human learning pointing out that pathways have been found that connect the executive and orienting networks of attention to the hippocampus. They review these functional-anatomic studies and lay out the specific predictions arising from them. They further suggest potential mechanisms for manipulating these pathways in mice including cutting-edge methods such as viral expression techniques. Buying is a self-reward process for some people.

In their ERP study, Ma et al. show that pain would enhance the neural activation for reward processing due to aggregated motivational salience. Intriguingly, one study suggests that about 20% of consumers suffer from pain at any one moment. Ma et al. hypothesize that the effects of pain may be revealed differently at early and later stages of the reward processing, evoking dynamic patterns of the FRN and P300. Results demonstrate that pain reduces the sensitivity to reward valence at an early stage and weakens the motivational salience at a later stage.

Han and Zhang investigate the different cognitive processes that are evoked by carnival and general promotions. After priming with promotion posters, the unknown e-commerce platform stimuli elicited larger P2 and N2 components than were observed after the presentation of general promotion posters. P2 may reflect the orienting of attention toward task-relevant target stimuli while N2 may reflect the process of cognitive control or the modulation of the detection of novel stimuli and the orienting of visual attention. For pilots to fly safely, continuous attention is essential.

In a ERP study, Zhang et al. employ an oddball paradigm to study the patterns of ERN, Pe and frequency band oscillations of volunteers and pilots in missions and analyze event-related potential, time-frequency, and brain function spectrum, extracting EEG indicators sensitive to error awarenes. They find that, in the 300–500 ms time window, the error awareness type was correlated with Pe amplitude and the error awareness of the pilots shows the same EEG sensitivity characteristics in flight as in the ground volunteer experiment, and the characteristic sensitivity value was higher than that of the ground participants.

In an eye tracking study, Zhou et al. seeks to identify the key determinant of the attentional mechanisms underlying time preference reversal. The behavioral results show that participants' time preferences and response times in the hybrid task were similar to those in the choice task but different from those in the bid task. Eye-tracking also reveals similar patterns, indicating that attention allocation, is similar but different from those in the bid task.

The study conducted by Wei et al. investigates the cognitive processes underlying the Ultimatum Game using eye-tracking technology. They also analyzed participants' DM behavior and evaluated their cognitive processes from a dual-system perspective Results showed that the estimated contributions of the two systems are uncorrelated and that they demonstrate a dissociated pattern of associations with third variables, such as reaction time (RT) and mean fixation duration (MFD). Their findings provide in review evidence for the independent contributions of preference for fairness (System 1) and self-interest maximizing (System 2) inclinations to UG decisions and shed light on the underlying processes.

Yang et al. undertake an eye-tracking study to explore the impact of an incentive strategy on decision confidence. They find that incentives do not affect intertemporal choice on the gain domain. By contrast, in the loss domain, subjects in the incentivized group were more likely to choose the larger-later options. This points to the disproportionate influence of loss stimuli in intertemporal choice.

Azulay et al. examine how empathy and stress measured by the Trier Social Stress Test may influence altruistic giving proxied by behavior in the Dictator Game. While acute stress by itself does not affect the level of altruistic giving, they find that individual differences in trait empathy moderate the effects of stress on giving. Elevations in stress-induced cortisol result in more generous giving, but only in individuals high in empathy. We note that attention is a prerequisite for the sentiment of empathy: when we are stressed or emotionally exhausted, our ability to pay attention may be impaired.

Chew and Ebstein begin with a description of how the frog catches flies instantly upon sensing them. Relying on retinal neurons which are activated by small, dark, and moving objects exclusively, the frog's “eye speaking directly to brain” circuitry obviates the need for higher cognitive processes accompanied by a complete loss of flexibility. This hard-wired sensory-perceptual process motivates a survey of early papers on the brain's bottom-up (stimulus driven) and top-down (goal directed) attention networks leading the authors to hone in on two pairs of neurotransmitters involved in valuation and attention processing—dopamine and serotonin (DA-5HT) and acetylcholine and norepinephrine (ACh-NE). Drawing on Zhong et al.'s (2009) model of the loss-gain differentiation in risk attitude based on DA-5HT tone, Chew and Ebstein present a tone-based hypothesis for the influence of ACh-NE on decision making under risk through the weight function in Chew's (1983) weighted utility theory. This sets the stage for inferring predictions linking ACh and NE tone to observable choice under risk which can be tested in placebo-controlled randomized controlled trials using pharmacological interventions.

## Author contributions

All authors listed have made a substantial, direct, and intellectual contribution to the work and approved it for publication.
